# Supplementary material for: Evaluation of a Community Intervention to Reduce Harmful Alcohol Consumption among Adult Population: A Study Protocol
Source: Int J Environ Res Public Health. 2022 Jul 18;19(14):8746. doi: 10.3390/ijerph19148746 (PMC9319312; doi:10.3390/ijerph19148746)
Supplement: Supplementary file 1 [file ijerph-19-08746-s001.zip › ijerph-1792541-supplementary.pdf]

Cuestionario sobre alcohol

¡Hola! El grupo de trabajo AcciOH (Acción comunitaria para reducir el consumo problemático de alcohol) está formado por profesionales de las entidades y recursos del territorio que trabajamos en acciones dirigidas a reducir el consumo problemático de alcohol. Con este cuestionario nos gustaría saber la opinión y conocimientos sobre el consumo de alcohol de las personas del barrio. Es muy sencillo y se responde rápidamente. ¡No te preocupes si crees que no conoces las respuestas, no te estamos evaluando!
La información que nos des será confidencial y anónima, solo la utilizaremos con fines estadísticos Tu participación es voluntaria.
¡Muchas gracias!

Doy mi consentimiento y autorizo al tratamiento de mis datos que voluntariamente cedo, atendiendo a las Leyes Orgánicas 2/2018, de 5 de diciembre de Protección de Datos Personales y Garantía de los Derechos Digitales y de la Ley 14/2007, de 3 julio, de Investigación Biomédica.

1. Sí
2. No

Sexo

1. Mujer
2. Hombre
3. No binario

Edad: ______

¿En qué barrio vives?

1. Turó de la Peira
2. Can Peguera
3. La Verneda i la Pau

¿Cuál es tu situación laboral actual?

1. Trabajas
2. Parado/a con subsidio/prestación
3. Parado/a sin subsidio/prestación
4. Trabajo en el hogar (ama de casa)
5. Estudiante
6. Incapacitado/a o con invalidez permanente
7. Jubilado/a
8. Otra situación, especifícala __________

¿Qué nivel de estudios has finalizado? (si estás estudiando actualmente, marca el último nivel que hayas acabado)

1. Sin estudios o primaria incompleta (sabe leer y escribir sin haber finalizado la educación primaria)
2. Estudios primarios completos (primaria, EGB, graduado escolar)
3. Estudios secundarios completos (ESO, Grado Medio, Grado Superior, PFI, PQPI, FP)
4. Estudios universitarios o superiores completos
5. Otros estudios, ¿cuáles? __________

Cuestionario sobre alcohol

A continuación, te haremos algunas preguntas relacionadas con conocimientos y consumo de alcohol

¿Crees que cualquier consumo de alcohol implica un riesgo para la salud?

1. Sí, cualquier consumo implica un riesgo
2. Sí, cuando se supera un cierto límite
3. No
4. No lo sé

¿Crees que el consumo de alcohol supone el mismo riesgo para hombres y mujeres?

1. Sí
2. No
3. No lo sé

El alcohol se mide en unidades de bebida, de manera que 1 unidad equivale a: 1 caña (250ml). Teniendo en cuenta esto, ¿a partir de cuantas unidades al día se considera un riesgo para la salud?

En mujeres el límite es...

1. 1 unidad
2. 2 unidades
3. 3 unidades
4. 4 unidades
5. 5 unidades
6. No lo sé

Y para hombres, ¿cuál es el límite?

1. 1 unidad
2. 2 unidades
3. 3 unidades
4. 4 unidades
5. 5 unidades
6. No lo sé

¿Cuántas bebidas alcohólicas crees que se tienen que beber en un período corto de tiempo para que se considere **consumo intensivo**? (por periodo corto de tiempo nos referimos a un intervalo aproximado de 2-4 horas)

	MUJERES	HOMBRES
Escribe el número de bebidas alcohólicas:	❑	❑

En qué grado te sientes capaz de decir “no” cuando alguien trata de hacerte beber alcohol (cerveza, vino o licor, …)

1. Nada capaz
2. Un poco capaz
3. Moderadamente capaz
4. Bastante capaz
5. Totalmente capaz

Cuestionario sobre alcohol

Ahora te haremos algunas preguntas relacionadas con tu consumo de alcohol (AUDIT 10 preg)

¿Con qué frecuencia bebes alguna bebida alcohólica?

1. Nunca
2. 1 o menos veces al mes
3. 2 o 4 veces al mes
4. 2 o 3 veces a la semana
5. 4 o más veces a la semana

¿Cuántas bebidas alcohólicas acostumbras a beber en un día en el que bebes?

1. 1 o 2
2. 3 o 4
3. 5 o 6
4. 7 a 9
5. 10 o más

¿Con qué frecuencia bebes 6 o más bebidas alcohólicas en un solo día?

1. Nunca
2. Menos de una vez al mes
3. Mensualmente
4. Semanalmente
5. A diario o casi a diario

¿Con qué frecuencia en el curso del último año te has encontrado que no podías parar de beber una vez que habías comenzado?

1. Nunca
2. Menos de una vez al mes
3. Mensualmente
4. Semanalmente
5. A diario o a casi a diario

¿Con qué frecuencia en el curso del último año no has podido realizar la actividad que te correspondía por haber bebido?

1. Nunca
2. Menos de una vez al mes
3. Mensualmente
4. Semanalmente
5. A diario o casi a diario

¿Con qué frecuencia en el curso del último año has necesitado beber en ayunas por la mañana para recuperarte de haber bebido mucho la noche anterior?

1. Nunca
2. Menos de una vez al mes
3. Mensualmente
4. Semanalmente
5. A diario o a casi a diario

¿Con qué frecuencia en el curso del último año has tenido remordimientos o sentimientos de culpa después de haber bebido?

1. Nunca

2. Menos de una vez al mes
3. Mensualmente
4. Semanalmente
5. A diario o casi a diario

¿Con qué frecuencia en el curso del último año has sido incapaz de recordar qué había pasado la noche anterior?

1. Nunca
2. Menos de una vez al mes
3. Mensualmente
4. Semanalmente
5. A diario o casi a diario

¿Tu o alguna otra persona se han hecho daño como consecuencia de que habías bebido?

1. No
2. Sí, pero no en el curso del último año
3. Sí, en el último año

¿Algún familiar, amigo, médico o profesional sanitario se ha preocupado por tu consumo de bebidas alcohólicas o te han sugerido que dejaras de beber?

1. No
2. Sí, pero no en el curso del último año
3. Sí, en el último año

Ya casi estamos! Una pregunta más!

El año que viene repetiremos este cuestionario, ¿podemos volver a contactarte? Las personas que acepten ser contactadas nuevamente participarán de un sorteo donde se entregarán 3 cheque-regalos, ¡por valor de 30 euros en tiendas del barrio!

1. Sí
2. No

Phone

Email Address

¡Ahora sí, ya hemos finalizado! ¡Muchas gracias!
¿Cómo crees que están tus conocimientos sobre el alcohol? Si quieres más información entra a la página https://drogues.gencat.cat
